# Activation of RHO-1 in cholinergic motor neurons competes with dopamine signalling to control locomotion

**DOI:** 10.1371/journal.pone.0204057

**Published:** 2018-09-21

**Authors:** Clara L. Essmann, Katie R. Ryan, Muna Elmi, Kimberley Bryon-Dodd, Andrew Porter, Andrew Vaughan, Rachel McMullan, Stephen Nurrish

**Affiliations:** MRC Laboratory for Molecular Cell Biology, University College London, London, United Kingdom; INSERM U869, FRANCE

## Abstract

The small GTPase RhoA plays a crucial role in the regulation of neuronal signalling to generate behaviour. In the developing nervous system RhoA is known to regulate the actin cytoskeleton, however the effectors of RhoA-signalling in adult neurons remain largely unidentified. We have previously shown that activation of the RhoA ortholog (RHO-1) in *C*. *elegans* cholinergic motor neurons triggers hyperactivity of these neurons and loopy locomotion with exaggerated body bends. This is achieved in part through increased diacylglycerol (DAG) levels and the recruitment of the synaptic vesicle protein UNC-13 to synaptic release sites, however other pathways remain to be identified. Dopamine, which is negatively regulated by the dopamine re-uptake transporter (DAT), has a central role in modulating locomotion in both humans and *C*. *elegans*. In this study we identify a new pathway in which RHO-1 regulates locomotory behaviour by repressing dopamine signalling, via DAT-1, linking these two pathways together. We observed an upregulation of *dat-1* expression when RHO-1 is activated and show that loss of DAT-1 inhibits the loopy locomotion phenotype caused by RHO-1 activation. Reducing dopamine signalling in *dat-1* mutants through mutations in genes involved in dopamine synthesis or in the dopamine receptor DOP-1 restores the ability of RHO-1 to trigger loopy locomotion in *dat-1* mutants. Taken together, we show that negative regulation of dopamine signalling via DAT-1 is necessary for the neuronal RHO-1 pathway to regulate locomotion.

## Introduction

The small Rho GTPase RhoA is a well-characterized regulator of the actin cytoskeleton [1,2]. In the nervous system RhoA acts on actin dynamics to regulate neuronal polarity [3], axon outgrowth [4], dendritic branching and synaptic connectivity [5]. RhoA has more recently been shown to play a role in neuronal activity by regulating endocytotic events [6], synaptic plasticity [7], and by enhancing learning and memory in adult mice [8]. Malfunctions in RhoA-signalling pathways are related to mental retardation phenotypes, which can be restored in the adult implicating new treatments for these disorders [9,10]. In *C*. *elegans* the single Rho-ortholog (RHO-1) is expressed throughout the nervous system [11] and is required for cell migration, cell shape regulation, and neurite outgrowth [12–14]. Our previous work together with the work of others demonstrates that RHO-1 acts via multiple pathways to regulate locomotion in adult animals [15–19]. Inhibition of endogenous RHO-1 signalling results in lethargic locomotion with shallow body bends [16,18,19], whilst activation of RHO-1 signalling triggers a neuronal hyperactivity and loopy locomotion associated with increased acetylcholine (ACh) release and exaggerated body bends [18]. RHO-1 regulates neuronal function downstream of a network of G-protein signalling pathways in cholinergic neurons [15–17], of which at least two regulate locomotion: RHO-1 regulates locomotion downstream of G_q_ via the activation of the NALCN ortholog NCA cation channels [16] and downstream of G_12_ through the regulation of diacylglycerol kinase (DGK-1) and the recruitment of the synaptic vesicle protein UNC-13 to release sites [18]. However, the evidence suggests that there are unidentified down stream targets of RHO-1 that are required for the regulation of locomotion [18].

Dopamine signalling controls mood, reward, motor control, and learning in humans [20]. It is negatively regulated by the dopamine transporter (DAT), which removes dopamine from the extracellular space reducing the concentration and spread of dopamine [21]. DAT is the target of legal (modafinil and Ritalin) and illegal (cocaine and amphetamines) psychostimulants [22], implicating its importance in regulating dopamine signalling. Polymorphisms in human DAT have been related to mental health disorders such as ADHD, drug abuse, bipolar disorder, Tourette syndrome, and autism [23–25]. Loss of function mutations of DAT have been associated with infantile parkinsonism-dystonia [26] and mutations in genes encoding DAT-associated proteins such as α-synuclein and Parkin are associated with inherited forms of Parkinson’s disease [27,28]. Pharmacological interventions targeting DATs through cocaine or amphetamine application result in the modulation of DAT function and lead to severe behavioural effects [29–31]. Therefore, understanding how DAT function, trafficking and gene expression are regulated has significance both biologically and sociologically. Many of these underlying mechanism have been studied [22,24,32–34], highlighting DAT as an integral component of the dopamine signalling pathway, which regulates behaviour.

Dopamine, which is negatively regulated by DAT, has a central role in modulating locomotion in both humans and *C*. *elegans* [35–37]. In *C*. *elegans*, dopamine signalling mutants fail to switch from swimming to crawling locomotion when they move from a liquid to a solid surface, they also fail to slow down when they encounter a bacterial lawn [37,38]. In contrast, increased dopamine signalling slows locomotion accompanied by shallow body bends [39–41]. Dopamine via DOP-3 receptors negatively modulates the activity of NCA channels to reduce locomotion whereas RHO-1 signalling activates them [16,17]. Thus, the dopamine and RHO-1 pathways have opposite effects on locomotion. The *C*. *elegans* DAT-1 is conserved with human DAT, shares trafficking mechanisms and has similar kinetics of dopamine reuptake and sensitivity to drugs [42–44]. Activation of RhoA has been shown to regulate DAT-internalization in mouse midbrain slices [31], and DAT-surface availability in *C*. *elegans* [43] cell autonomously linking RhoA to DAT function. In this study, we show that *dat*-1 is required for RHO-1 to modulate locomotion *in vivo*. We propose a model whereby enhanced RHO-1 signalling in cholinergic motor neurons leads to a cell non-autonomous transcriptional upregulation of *dat-1* in dopaminergic neurons. This decreases the dopaminergic signalling that normally acts to reduce locomotion and enables RHO-1 signalling to drive locomotion. Our data suggest that the neuronal RHO-1 pathway regulates *dat-1* gene expression to fine-tune locomotory behaviour.

## Results

### RHO-1 signalling regulates locomotion

**Fig 1:**
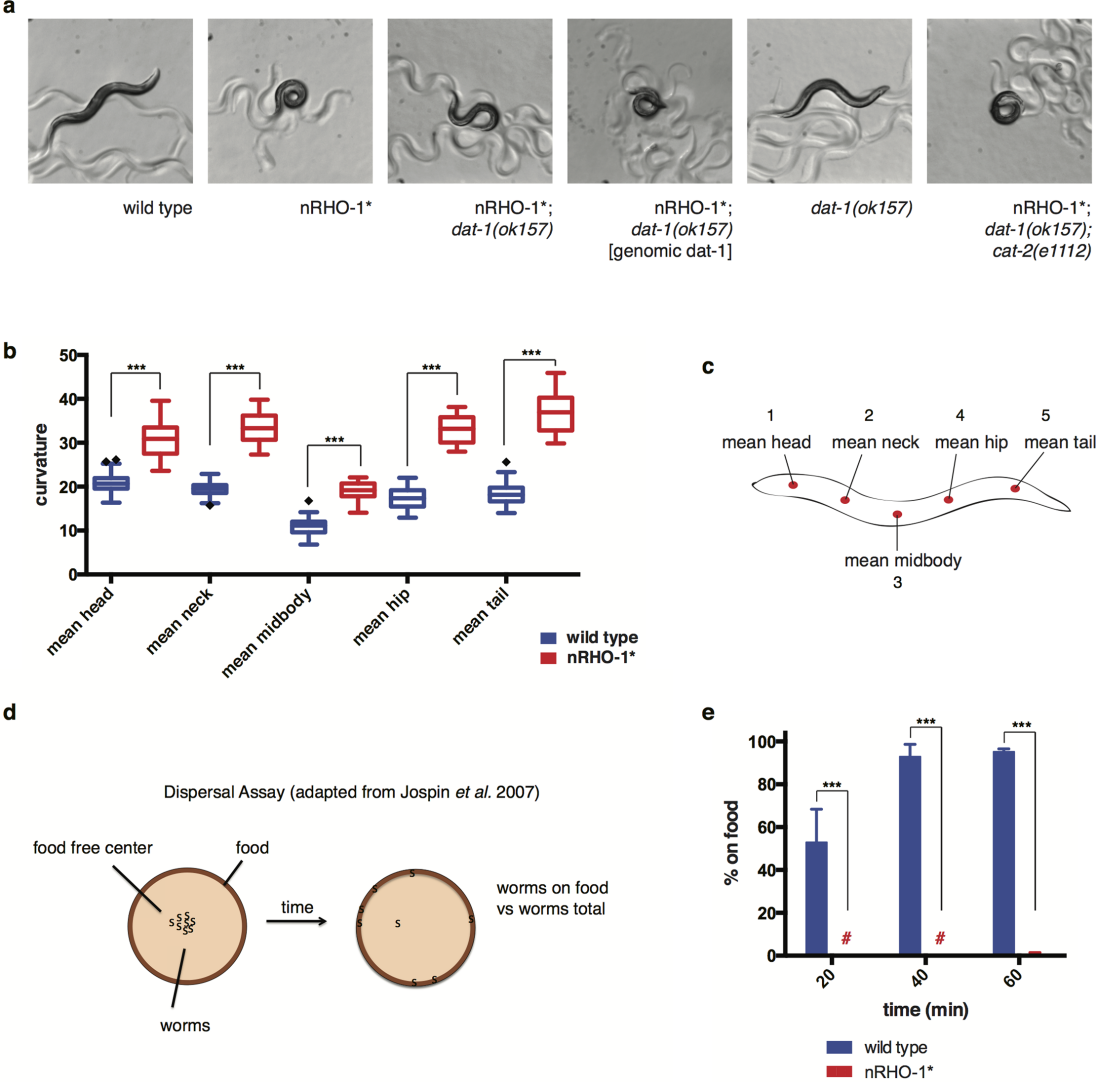
nRHO-1* expression alters locomotion phenotypes. **a** Representative images of control strains (wild type, *dat-1(ok157))* and different nRHO-1* animals showing characteristic body curvature and motion tracks on a culture dish (8x magnification). **b** Worm tracking data of body curvature at 5 body points within the worm (as shown in c) comparing control animals (wild type) to nRHO-1* animals. Statistical comparisons were performed using one-way ANOVA and presented as mean curvature ± SEM with Tukey’s multiple test correction. Diamonds indicate outliers. Statistical significance indicated as (***P ≤ 0.001), n = 70 and 32 animals respectively. **c** Schematic of a *C*. *elegans*. Reference points to measure body curvature using worm tracker terminology and indicated as red dots.**d** Schematic presentation of dispersal assay as described in materials and methods. **e** Dispersal assay as described in materials and methods. Genotypes are color-coded as indicated below the panel. Hash keys represent data points ≤ 1%. Data presented as the mean percentage of animals on food at indicated time points ± SEM. Statistical comparisons were performed using two-way ANOVA with Tukey’s multiple test correction. Statistical significance indicated as (***P ≤ 0.001), n = 3 experiments (~200 animals per assay).

Previously we have shown that RHO-1 signalling in adult cholinergic neurons is required for normal locomotion [18,45]. Reducing RHO-1 signalling, by expressing the RHO-1 inhibitor C3 transferase in adult cholinergic neurons or using the Rho GEF mutant *unc-73(ce362)*, results in lethargic locomotion characterized by flat, shallow body bends [18,19] while expressing the constitutively active RHO-1 (RHO-1 (G14V)) mutant from the *unc-17* promoter in cholinergic neurons (nRHO-1*) drives hyperactive locomotion, best characterized by exaggerated loopy body bends (Fig 1A, S2 Movie) [18]. For a more detailed analysis of the nRHO-1* mediated loopy locomotion behaviour we quantified body curvature using the Worm Tracker 2.0.4 system and the Worm Analysis Tool Box version 3 [46]. The degree of curvature of hyperactive loopy nRHO-1* animals compared to wild type animals was significantly increased at each of the five equally spaced points along the body (all p< = 0.0001) (Fig 1B and 1C, S1 and S2 Movies), indicating that nRHO-1* increases curvature along the entire body (Fig 1C). For clarity one point on the worm, mean hip (point 4), was chosen to represent alterations in curvature on subsequent strains (See Material and Methods). Given that nRHO-1* animals have altered body curvature we employed a second locomotion assay, the dispersal assay, to determine the effect of this change on the animal’s ability to cover distance over time. Animals are placed in the centre of a large NGM plate that has a narrow ring of food around the outside edge. The number of animals that reach the ring of food, at given time points, are scored as a percentage of the total number of animals on the plate [47] (Fig 1D). We observed that 95.2% ±0.8 of the wild type animals reached the food after 60 minutes while within this time only 0.5% ±0.5 of nRHO-1* animals did so (p< = 0.0001) (Fig 1E). Approximately 50% of the nRHO-1* animals reached the food when plates were left overnight (data not shown) suggesting that despite being loopy nRHO-1* hyperactive animals are still able to direct their locomotion to a certain extent, although at a far slower rate when compared to wild type animals.

### *dat-1* mutations suppress nRHO-1* mediated changes in locomotion

**Fig 2:**
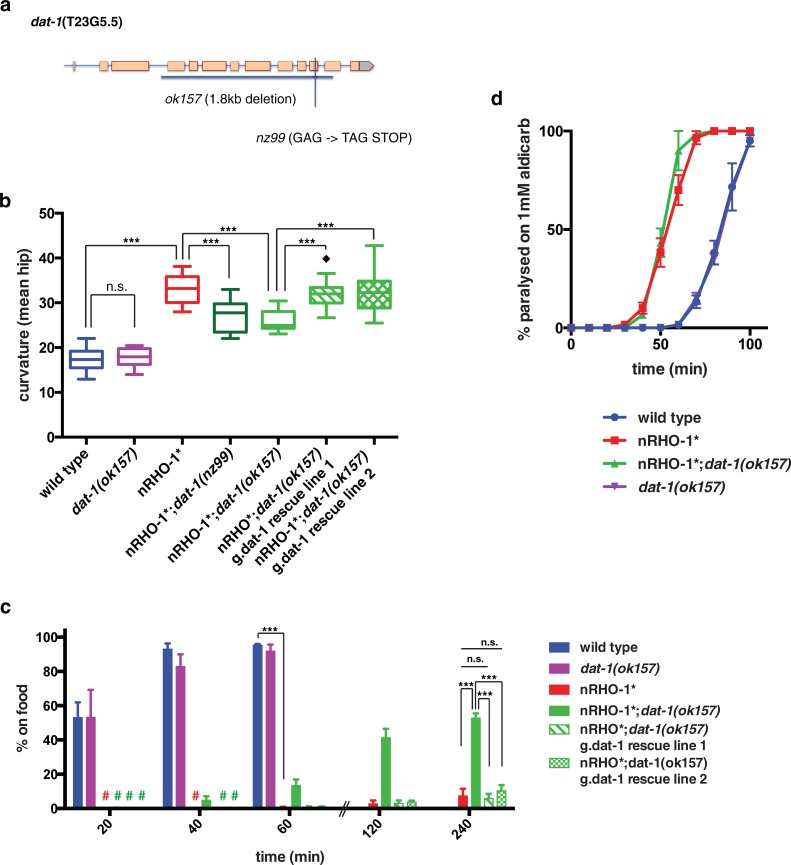
A *dat-1* mutation suppresses nRHO-1* locomotion phenotypes. **a** Schematic of the *dat-1* gene with alleles *ok157* and *nz99* indicated. **b** Worm tracking data of body curvature measurements of control animals (wild type, *dat-1(ok157)*), nRHO-1* animals, nRHO-1* suppressor strains (nRHO-1*;*dat-1(nz99)* and nRHO-1*;*dat-1(ok157)*) and rescue lines. Statistical comparisons were performed using one-way ANOVA and presented as mean hip ± SEM with Tukey’s multiple test correction. Diamonds indicate outliers. Statistical significance indicated as (***P ≤ 0.001, n.s. = not significant), n = 70, 14, 32, 19, 15, 18, and 17 animals respectively.**c** Dispersal assay as described in materials and methods. Genotypes are color-coded as indicated on the right side of the panel. Hash keys represent data points ≤ 1%. Data presented as the mean percentage of animals on food at indicated time points ± SEM. Statistical comparisons were performed using two-way ANOVA with Tukey’s multiple test correction. Statistical significance indicated as (***P ≤ 0.001, n.s. = not significant), n = 3 experiments (~200 animals per assay).**d** Aldicarb assay as described in materials and methods. Control strains (wild type, *dat-1(ok157))*, nRHO-1* animals and nRHO-1*;*dat-1(ok157)* double mutant assayed on 1mM Aldicarb. Genotypes are color-coded as indicated below the panel. Data presented as mean percentage of paralyzed animals scored every 10 minutes up to 100 min ± SEM, n = 3 (>30 animals per assay).

Our previous work together with the recent work of others [16,18] demonstrates that RHO-1 acts via multiple pathways to regulate locomotion. One of these pathways involves RHO-1 negatively regulating diacylglycerol kinase (DGK-1) to increase diacylglycerol (DAG) levels at release sites resulting in the recruitment of UNC-13. However, other DGK-1 independent pathways acting downstream of RHO-1 remain to be identified [18]. To identify these other components of neuronal RHO-1 signalling pathways we performed a forward genetic screen for mutations that suppressed nRHO-1* mediated increases in curvature. The original suppressor screen strain had both versions of active RHO-1, nRHO-1* and heat shock hs::RHO-1*, only alleles supressing both were chosen to be suppressor mutants. One mutant, *nz99*, showed a significant decrease in curvature from 33.1° ±0.5 in nRHO-1* to 27.1° ±0.8 in nRHO-1*;*nz99* animals (p< = 0.0001) (Fig 2B). Whole genome sequencing of the nRHO-1*;*nz99* strain identified 42 mutations predicted to alter protein coding (S1 Table). Among these only one nonsense mutation was identified, which encoded a GAG->TAG (Glu->stop) change in the dopamine transporter DAT-1 (Fig 2A). The mutation in *dat-1* was of significant interest as changes in dopamine signalling are known to affect locomotion [48]. Therefore, we sought to confirm whether decreasing DAT-1 could suppress nRHO-1* using a canonical *dat-1* allele, *ok157* (Fig 2A). Like the suppressor mutation *nz99 dat-1(ok157)* significantly suppressed nRHO-1*-mediated increased curvature from 33.1° ±0.5 in nRHO-1* to 25.8° ±0.6 in nRHO-1*;*dat-1(ok157)* (p< = 0.0001) (Figs 1A and 2B, S3 Movie). This *dat-1(ok157*) allele, like *nz99*, was also able to supress the loopy locomotion in our heat shock activated hs::RHO-1* strain (Figure a in S1 Fig). Previously, Yemini et al reported that *dat-1(ok157)* animals have alterations in a number of locomotory aspects [49]. We analysed mean curvature of the hip as well as the other 4 body points and observed no significant difference between the *dat-1(ok157)* and wild type animals (Figs 1C and 2B, Figure b in S1 Fig, S1 and S4 Movies). Similarly, we observed no significant difference in the dispersal assay between *dat-1(ok157)* and wild type animals (Fig 2C) indicating that *dat-1(ok157)* animals are able to co-ordinate their locomotion to the same extent as wild type animals. However, the *dat-1(ok157)* mutation significantly suppressed the dispersal phenotype of nRHO-1*. After 4 hours 52.7% ±2.8 of the nRHO-1*;*dat-1(ok157)* animals reached the food compared to only 7.3% ±4.2 of nRHO-1* animals (p< = 0.0001) (Fig 2C). Approximately 100% of the nRHO-1*;*dat-1(ok157)* animals reached the food when plates were left overnight (data not shown). For all further experiments we used the *dat-1(ok157)* allele instead of the *nz99* strain, as this allele has been widely reported in the literature, and strains in which *dat-1(ok157)* has been extensively backcrossed are available.

Although *dat-1(ok157)* significantly suppressed the increase in curvature and failure to disperse at 4 hours observed in nRHO-1* animals, it did not fully restore the nRHO-1* levels of curvature (17.4° ±0.3 in wild type compared to 25.8° ±0.6 in nRHO-1*;*dat-1(ok157)*, p< = 0.0001) and dispersal (100% ±0.0 wild type at 4 hours compared to 52.7% ±2.8 nRHO-1*;*dat-1(ok157)*, p< = 0.0001) to that of wild type animals (Fig 2B and 2C, S1 and S3 Movies). This is consistent with previous data indicating that RHO-1 signals via multiple effectors to regulate locomotion [16,18]. We performed rescue experiments to confirm whether the *dat-1(ok157)* mutation was indeed a suppressor of nRHO-1*. Two independent lines expressing a genomic fragment containing wild type *dat-1* restored the ability of increased RHO-1 signalling to increase curvature and to prevent dispersal in nRHO-1*;*dat-1(ok157)* animals. For example, the curvature of rescue (line 1) was 32.5° ±0.1 compared to 25.8° ±1.9 in nRHO-1*;*dat-1(ok157)* and not significantly different from 33.1° ±0.5 in nRHO-1* (p< = 0.0001; p = 0.883). The dispersal of rescue (line 1) at 4 hours was 5.8% ±2.7 compared to 52.7% ±2.8 in nRHO-1*;*dat-1(ok157)* (p< = 0.0001) and not significantly different from 7.3% ±4.2 in nRHO-1* (p> = 0.999) (Figs 1A, 1B and 2C, S5 Movie). These data indicate that the ability of RHO-1 signalling to drive locomotion is compromised in *dat-1* mutants.

### *dat-1* is not required for nRHO-1* increased ACh release

Constitutively active RHO-1 signalling increases the release of ACh onto body wall muscles, which leads to a faster rate of paralysis in the presence of the acetylcholinesterase inhibitor aldicarb (Fig 2D) [18]. We have previously assumed that the hyperactive loopy locomotion of nRHO-1* animals is a result of increased ACh release hyper-activating muscles. Interestingly, we found that *dat-1(ok157)* has no impact on the increased ACh release (hyperactive phenotype) observed in constitutively active RHO-1 animals, as the nRHO-1*;*dat-1(ok157)* double mutants remain hypersensitive to aldicarb despite being less loopy. For example, at 50 minutes, 0% ± 0.0 of wild type animals were paralysed compared to 38.3% ±7.3 of nRHO-1* (p< = 0.0001), or to 43.3% ±7.3 of nRHO-1*;*dat-1(ok157)* (p< = 0.0001) (Fig 2D). These data indicate that increased ACh release at the neuromuscular junction is not solely responsible for the increase in body curvature and dispersal phenotypes in animals with enhanced RHO-1 signalling. Our data suggest additional DAT-1 dependent pathways are also required to cause the observed changes in locomotory behaviour.

### Dopamine suppresses the locomotion phenotypes of constitutively active RHO-1 signalling

**Fig 3:**
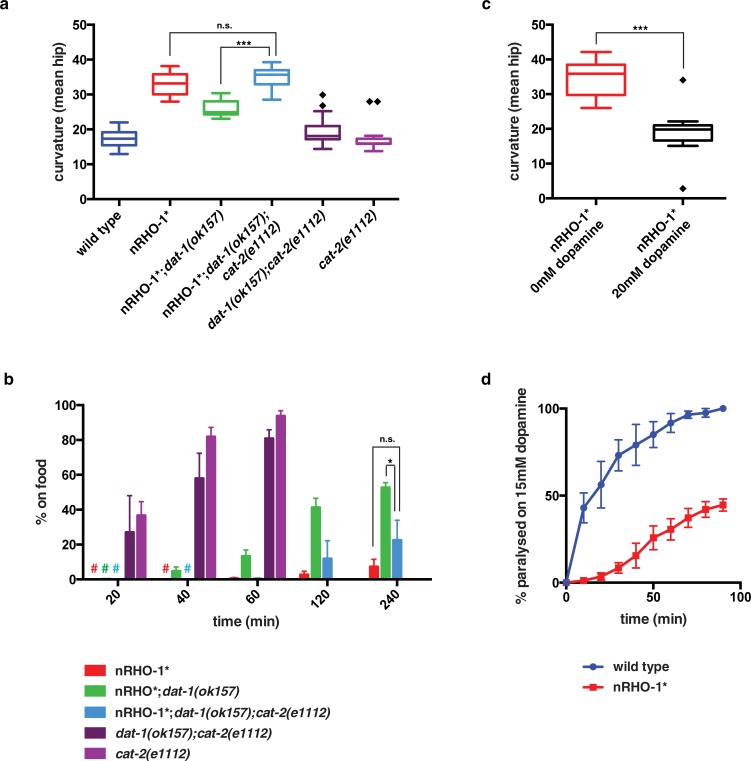
Dopamine antagonizes nRHO-1*. **a** Worm tracking data of body curvature of control animals (wild type, nRHO-1*, *dat-1(ok157)*, nRHO-1*;*dat-1(ok157)* the same data used in Fig 2B), dopamine synthesis mutants (*cat-2* (e1112), *dat-1(ok157)*;cat-1(e1112)) and nRHO-1*;*dat-1(ok157)*;*cat-2*(e1112) triple mutants. Statistical comparisons were performed using one-way ANOVA and presented as mean hip ± SEM with Tukey’s multiple test correction. Diamonds indicate outliers. Statistical significance indicated as (***P ≤ 0.001, n.s. = not significant), n = 70, 32, 15, 18, 30, and 14 animals respectively. **b** Dispersal assay as described in materials and methods. Genotypes are color-coded as indicated below the panel. Hash keys represent data points ≤ 1%. Data presented as the mean percentage of animals on food at indicated time points ± SEM. Statistical comparisons were performed using two-way ANOVA with Tukey’s multiple test correction. Statistical significance indicated as (*P ≤ 0.05–0.01, n.s. = not significant), n = 3 experiments (~200 animals per assay). **c** Worm tracking data of body curvature of nRHO-1* animals after 20 minutes on plates containing 0 mM or 20 mM dopamine. Presented as mean hip ± SEM. Statistical comparisons were performed using an unpaired two-tailed t-test. Diamonds indicate outliers. Statistical significance indicated as (***P ≤ 0.001), n = 16 and 25 animals respectively. **d** Dopamine assay as described in materials and methods. Control strains (wild type) and nRHO-1* animals assayed on 15 mM dopamine. Genotypes are color-coded as indicated below the panel. Data presented as mean percentage of paralyzed animals scored every 10 minutes up to 100 min ± SEM, n = 3 experiments (~30 animals per assay).

DATs negatively regulate dopamine signalling by removing dopamine from the extracellular space, thus DAT reduces the concentration and the spread of dopamine released from the dopaminergic neurons [21]. We predicted that *dat-1 (ok157)* animals, which lack DAT-1, could potentially have increased extracellular dopamine levels and dopamine signalling. We used two approaches to examine these possibilities. Firstly, if increased dopamine signalling is required to suppress nRHO-1* locomotion then this suppression would be compromised in mutants defective for dopamine synthesis such as *cat-2*, which encodes a tyrosine hydroxylase, a rate-limiting enzyme required for dopamine synthesis [50]. Our results show that the *cat-2(e1112)* mutation restores curvature and dispersal in nRHO-1*;*dat-1(ok157)* animals back to similar levels observed in nRHO-1* animals (curvature of nRHO-1*;*dat-1(ok157)*;*cat-2(e1112)* 34.9° ±0.7 compared to nRHO-1*;*dat-1(ok157)* 25.8° ±0.6, p< = 0.0001 or nRHO-1* 33.1° ±0.5, p = 0.26 and dispersal at 4 hours, 22.5% ±11.4 compared to 52.7% ±2.8, p< = 0.01 or nRHO-1* 7.3% ±4.2, p = 0.53) (Figs 1A, 3A and 3B, S6 Movie). Secondly, we exposed animals to exogenous dopamine in order to analyse the effects of increased dopamine levels on the animal’s locomotory behaviour. Exposure to dopamine significantly suppressed the increased curvature phenotype of nRHO-1* animals from 34.4° ±1.2 on 0mM dopamine to 18.9° ±1.0 on 20mM dopamine (p< = 0.0001) (Fig 3C, S7 Movie). In other words, exogenous dopamine exposure phenocopies the suppressive effects of the *dat-1* mutation. Our data indicate that it is an increase in extracellular dopamine in *dat-1* mutants that suppresses the nRHO-1* hyperactive loopy locomotion phenotype. Interestingly, 15mM exogenous dopamine paralyzes 85.0% ±7.4 of wild type animals at 50 minutes compared to only 25.8% ±6.8 of nRHO-1* animals (p< = 0.0001) (Fig 3B). These data indicate that dopamine and nRHO-1* co-suppress each other’s locomotion phenotypes, suggesting that these pathways antagonistically regulate locomotion.

### Dopamine acts through the DOP-1 receptor to antagonize locomotion

**Fig 4:**
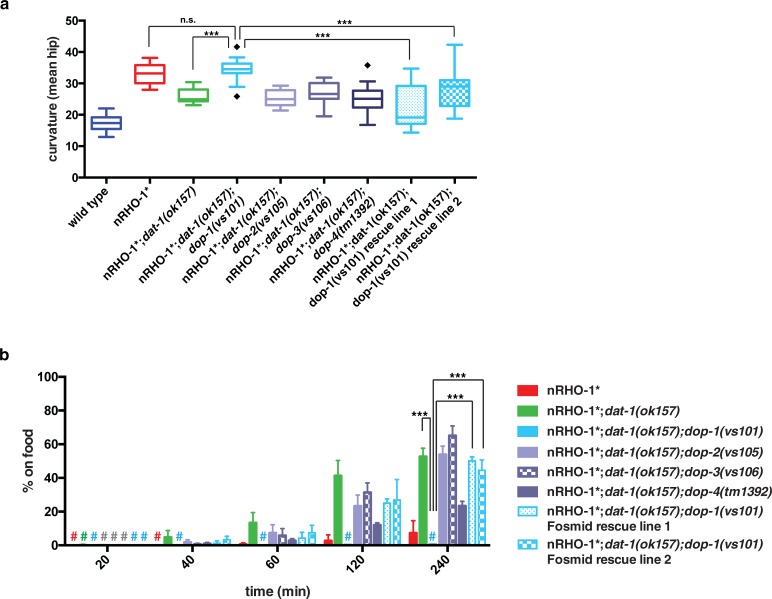
Suppression of nRHO-1* loopy locomotion mediated via DOP-1. **a** Worm tracking data of body curvature of control animals (wild type, *dat-1(ok157)*, nRHO-1*;*dat-1(ok157)* the same data used in Figs 2B and 3A), four dopamine receptor nRHO-1*;*dat-1(ok157)* triple mutants and two independent rescue lines expressing the dop-1 containing fosmid WRM069cF02. Statistical comparisons were performed using one-way ANOVA and presented as mean hip ± SEM with Tukey’s multiple test correction. Diamonds indicate outliers. Statistical significance indicated as (***P ≤ 0.001, n.s. = not significant), n = 70, 32, 15, 17, 16, 20, 16, 21, and 17 animals respectively. **b** Dispersal assay as described in materials and methods. Genotypes are color-coded as indicated right of the panel. Hash keys represent data points ≤ 1%. Data presented as the mean percentage of animals on food at indicated time points ± SEM. Statistical comparisons were performed using two-way ANOVA with Tukey’s multiple test correction. Statistical significance indicated as (***P ≤ 0.001), n = 3 experiments (~200 animals per assay).

To date, *C*. *elegans* have four confirmed dopamine receptors (DOP-1, -2, -3 and DOP-4) whose homology to mammalian receptors can be used to classify them into D1-like (DOP-1 and DOP-4) and D2-like (DOP-2 and DOP-3) dopamine receptors [48]. To determine which, if any, of the four receptors mediates the suppression of hyperactive locomotion in nRHO-1*;*dat-1(ok157)* animals we made nRHO-1*;*dat-1(ok157)* triple mutants with each *dop* mutant. DOP-1 may play a role as the nRHO-1*;*dat-1(ok157)*;*dop-1(vs101)* animals had increased curvature of 34.5° ±0.9 indistinguishable from that of nRHO-1* single mutants with 33.1° ±0.5 (p = 0.9374) (Fig 4A, S8 Movie). These triple mutant animals also failed to disperse with 0.6% ±0.2 at 4 hours similar to nRHO-1* with 7.3% ±4.2 (p = 0.7513) (Fig 4B). In two independent lines transgenic animals with a fosmid containing the wild type *dop-1* gene restored the suppressive ability of the *dat-1* mutation in nRHO-1*;*dat-1(ok157)*;*dop-1(vs101)* animals. These animals have decreased curvature, for example rescue line 1 has 21.9° ±1.3 compared to 34.5° ±0.9 of nRHO-1*;*dat-1(ok157)*;*dop-1(vs101)* triple (p< = 0.0001), and has increased dispersal at 4 hours with 50.1% ±1.4 compared to 0.6% ±0.2 of nRHO-1*;*dat-1(ok157)*;*dop-1(vs101)* triple (p< = 0.0001), hence it is now similar to nRHO-1*;*dat-1 (ok157)* mutants (Fig 4A and 4B). Mutations in *dop-4* had a partial effect only on the dispersal behaviour of the nRHO-1*;*dat-1(ok157)*;*dop-4(tm1392)* triple mutants (Fig 4B), however, these animals appeared to be very sick which probably affected their locomotion (C.E. personal observation). *dat-1(ok157)*;*dop-4(tm1392)* double mutants had a dispersal phenotype similar to *dat-1(ok157)* (Figure b in S2 Fig). Attempts to rescue *dop-4* in the nRHO-1*;*dat-1(ok157)*;*dop-4(tm1392)* animals were unsuccessful. Therefore it is unclear if DOP-4 plays a specific role in modulating the dopamine signal that antagonizes RHO-1 signalling. Mutations in *dop-2* or *dop-3* had no effect on the locomotion of nRHO-1*;*dat-1(ok157)* animals indicating that *dop-2* and *dop-3* had no impact on *dat-1* suppression of nRHO-1* locomotion (Fig 4A and 4B). The control animals, each of the single *dop* mutant animals (in a wild type background) and their doubles with *dat-1(ok157)* had wild type locomotion (Figure a in S2 Fig). Taken together, these data indicate that mutations in *dat-1* lead to increased extracellular dopamine that acts primarily via DOP-1 to antagonize the locomotion phenotypes of nRHO-1* animals.

### RHO-1 signalling regulates *dat-1* expression

**Fig 5:**
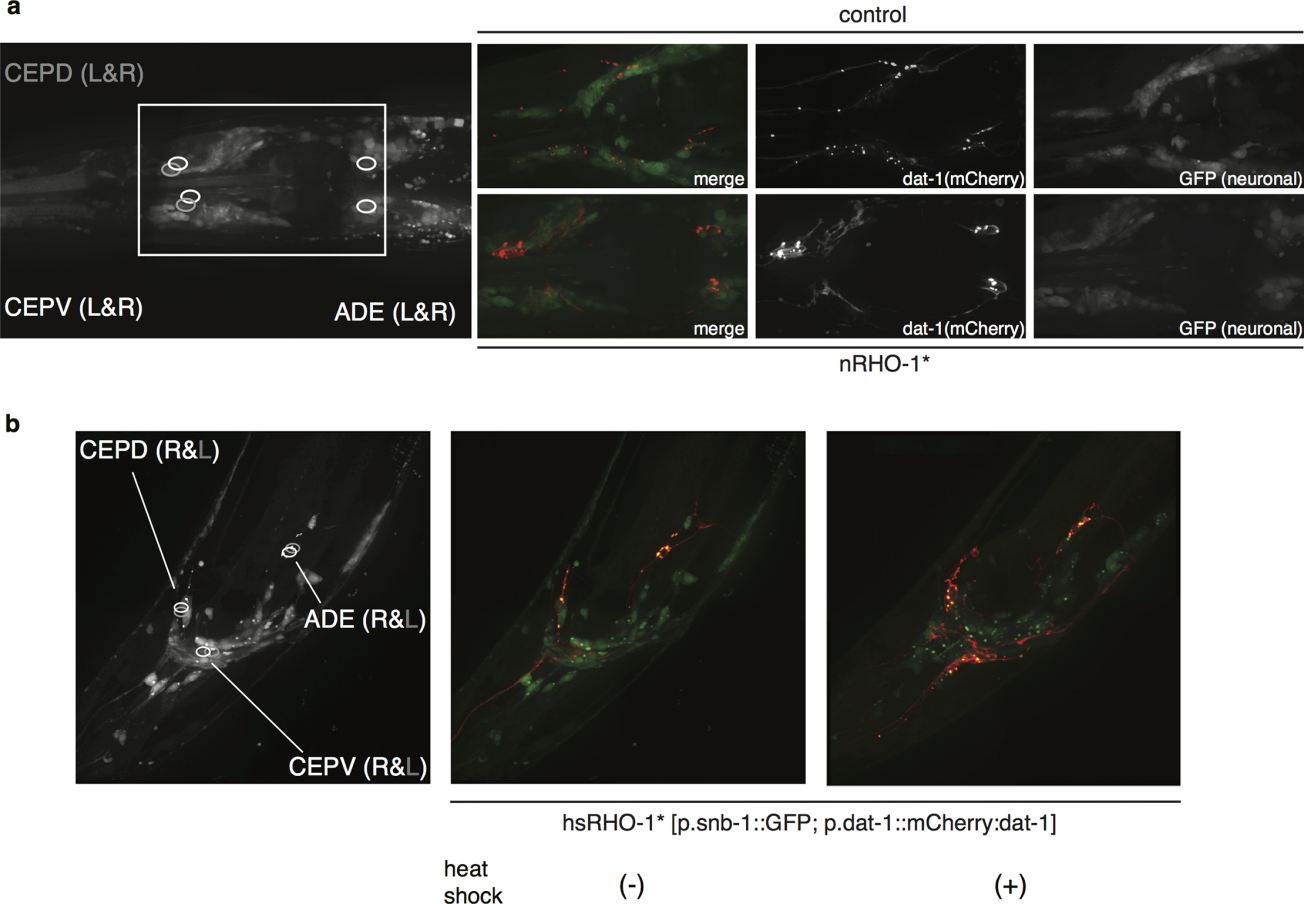
nRHO-1* increases levels of DAT-1 protein. **a** Representative head images of animals expressing *dat-1* fused to mCherry under its endogenous promoter (indicated in red), and a general neuronal maker, GFP expressed from the synaptobrevin promoter (indicated in green). Three pairs of dopaminergic head neurons indicated by circles in the left panel. Wild type strain (upper pictures), nRHO-1* animals (lower pictures). Images taken at 100x magnification as described in methods. **b** Representative head images of heat shock inducible RHO-1* (hsRHO-1*) animals expressing *dat-1* fused to mCherry under its endogenous promoter (p.*dat-1*::mCherry:*dat-1*) indicated in red, and a general neuronal maker, GFP expressed from the synaptobrevin promoter (p.*snb-1*::GFP), indicated in green. Three pairs of dopaminergic head neurons indicated by circles in the left panel. No heat shock (middle panel), after heat shock (right panel). Images taken at 100x magnification as described in methods.

**Fig 6:**
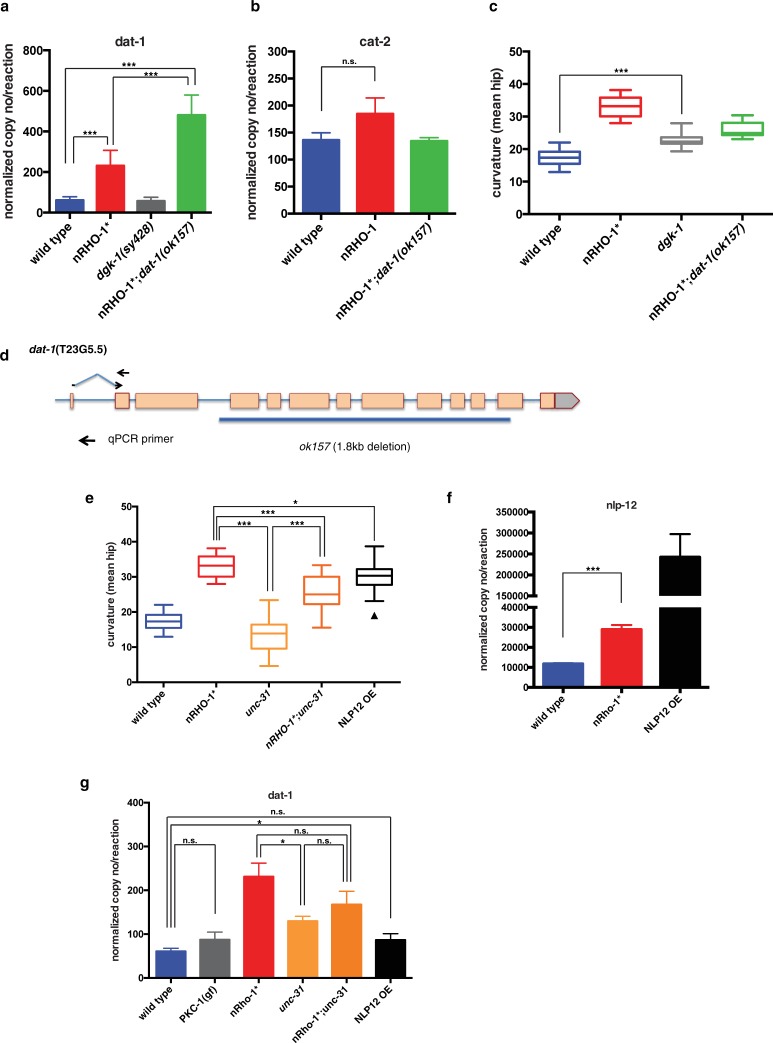
nRHO-1* increases transcription levels of *dat-1*. **a and b** Transcription levels of *dat-1* or *cat-2* in nRHO-1* single and nRHO-1*;*dat-1(ok157)* double mutants compared to control strains (wild type, *dgk-1(sy428*)) determined by quantitative PCR. Data presented as mean normalized copy number per reaction ± SEM. Statistical comparisons were performed using one-way ANOVA with Tukey’s multiple test correction. Statistical significance indicated as (***P ≤ 0.001, n.s. = not significant), n = 6, 6, 3, 4 for a and n = 5, 5, 4 for b independent samples for each genotype respectively. **c** Worm tracking data of body curvature of control animals (wild type, nRHO-1*, nRHO-1*;*dat-1(ok157)* the same data used in Figs 2B, 3A and 4A*)*, or *dgk-1(sy428)* animals. Statistical comparisons were performed using one-way ANOVA and presented as mean hip ± SEM with Tukey’s multiple test correction. Statistical significance indicated as (***P ≤ 0.001), n = 70, 32, 14, and 15 animals respectively.**d** Schematic of the *dat-1* gene with the *ok157* allele indicated. qPCR primer set for *dat-1* within first and second exon shown as arrows.**e** Worm tracking data of body curvature of control animals (wild type, nRHO-1* the same data used in Figs 2B, 3A, 4A and 6C), *unc-31(e928)*, nRHO-1*;*unc-31(e928)* and NLP12 OE animals. Statistical comparisons were performed using one-way ANOVA and presented as mean hip ± SEM with Tukey’s multiple test correction. Triangles indicate outliers. Statistical significance indicated as (*P ≤ 0.05, ***P ≤ 0.001), n = 70, 32, 12, 16 and 14 animals respectively.**f** Transcription levels of *nlp-12* in nRHO-1* compared to control (wild type) and NLP12 OE strains determined by quantitative PCR. Data presented as mean normalized copy number per reaction ± SEM. Statistical comparisons were performed using one-way ANOVA with Tukey’s multiple test correction. Statistical significance indicated as (***P ≤ 0.001) n = 6, 6 and 3 independent samples for each genotype respectively. **g** Transcription levels of *dat-1* in control (wild type, nRHO-1* the same data used in Fig 6A) compared to *pkc-1(gf)*, *unc-31(e928)*, nRHO-1*;*unc-31(e928)* and NLP12 OE strains determined by quantitative PCR. Data presented as mean normalized copy number per reaction ± SEM. Statistical comparisons were performed using one-way ANOVA with Tukey’s multiple test correction. Statistical significance indicated as (*P ≤ 0.05, n.s. = not significant), n = 6 for all apart from n = 3 for NLP12 OE independent samples for each genotype.

Our data suggest that RHO-1 and dopamine signalling antagonistically regulate locomotion, but by what mechanism does RHO-1 signalling reduce dopamine signalling to drive locomotion? DAT-1 is the major negative regulator of dopamine signalling and is required for RHO-1 mediated changes in locomotion. Our data indicates that nRHO-1* animals paralyze at a slower rate compared to wild type animals when exposed to exogenous dopamine (Fig 3D). We investigated whether the expression or localisation of DAT-1 was altered in nRHO-1* animals. We first created a p.*dat-1*::mCherry::DAT-1 translational fusion using the same genomic fragment that we used for the *dat-1* rescue (Figs 1A and 2B and 2C). mCherry signals were detected in the four pairs of dopaminergic cells (PDE, ADE, CEPD and CEPV) in both wild type and nRHO-1* animals suggesting the expression pattern of *dat-1* was unchanged by nRHO-1* [41,51]. In nRHO-1* animals we saw an obvious increase in the fluorescence of the p.*dat-1*::mCherry::DAT-1 reporter compared to wild type animals expressing the same transgene suggesting that RHO-1 increases mCherry::DAT-1 protein levels (Fig 5A). Moreover, this increase could be induced in adult animals by expressing constitutively active RHO-1 from a heatshock-inducible promoter (Fig 5B). In contrast, expression of a co-injected pan-neuronal GFP reporter (p.*snb-1*::GFP) showed no obvious change (Fig 5A and 5B). We then tested our observation, that *dat-1* expression is increased in the presence of nRHO-1* by analysing the mRNA level of the endogenous *dat-1* gene with qPCR. *dat-1* mRNA levels were almost four times higher in nRHO-1* animals with 231.1 ±30.9 copy no/reaction compared to wild type with 60.8 ±7.0 copy no/reaction (p< = 0.0001) (Fig 6A). *dat-1* is expressed exclusively in dopaminergic cells, an expression pattern shared with *cat-2*, which is required for dopamine synthesis [52]. *cat-2* expression however, was not significantly altered by constitutively active nRHO-1* (p = 0.998) (Fig 6B). We also analysed the expression levels of the 4 dopamine receptors (*dop-1-4)*, and a general neuronal gene synaptobrevin (*snb-1*) to exclude the possibility that nRHO-1* was affecting global transcription. We observed no significant change in expression of *dop-1-4* or *snb-1* in nRHO-1* animals compared to wild type animals (Figure a-e in S3 Fig). These data indicate that RHO-1 signalling regulates *dat-1* expression in particular, and not generally the expression of components of the dopamine signalling pathway such as *dop-1-4* or *cat-2*. However, the transcription of other genes not tested here may also be altered in nRHO-1* animals. The constitutively active RHO-1 construct is under the *unc-17* promoter which drives expression in cholinergic neurons [17,18], therefore nRHO-1* and *dat-1’s* expression pattern best fits a cell non-automonous regulation model.

### Increased *dat-1* expression is not a consequence of increased body curvature, or increased ACh, or neuropeptide release

We investigated whether the increase in body curvature or the presence of DAT-1 protein itself could cause the increase in *dat-1* expression in nRHO-1* animals. Although the *dat-1(ok157)* mutant fails to produce functional DAT-1 protein, the *dat-1* gene retains the promoter and transcriptional start sites. Using primers 5’ of the *dat-1(ok157)* deletion (Fig 6D) we detected an even higher increase of *dat-1* mRNA in nRHO-1*;*dat-1(ok157)* animals of 480.4 ±49.5 copy no/reaction than we did in nRHO-1* animals with 231.1 ±30.9 copy no/reaction (p< = 0.0001) (Fig 6A). As nRHO-1*;*dat-1(ok157)* animals lack both functional DAT-1 protein and exaggerated body bends we concluded neither of these are required for nRHO-1* mediated increases in *dat-1* transcription. We have previously shown that neuronal RHO-1 negatively regulates the diacylglycerol kinase DGK-1 [18]. *dgk-1* mutations partly mimic constitutively active RHO-1* animals as they have elevated ACh release [18,53] and increase their body curvature to a level intermediate between that of wild type and nRHO-1* animals [53] and similar to that of nRHO-1*;*dat-1(ok157)* animals (*dgk-1* 22.7° ±0.6 compared to nRHO-1*;*dat-1(ok157)* 25.8° ±0.6, p = 0.0042) (Fig 6C). However, unlike either nRHO-1* or nRHO-1*;*dat-1(ok157)* animals *dgk-1* mutants do not increase *dat-1* expression (57.1 ±10.9 copy no/reaction compared to wild type 60.8 ±7.0, p> = 0.9999) (Fig 6A). These data indicate that the upregulation of *dat-1* expression represents a new RHO-1 neuronal signalling pathway acting independently of DGK-1, body curvature and elevated ACh release.

In addition to neurotransmitters, neuropeptides play a key role in regulating locomotion [54–56]. We hypothesised that neuropeptides may be involved in our nRHO-1* predicted cell non-autonomous regulation of *dat-1* expression since a number of studies have shown that neuropeptides act in a cell non-autonomous manner [57] and can regulate gene expression [58,59]. Bhattcharya *et al*, showed that the neuropeptide NLP-12 modulates dopamine signalling via DOP-1 and that over expression of *nlp-12* (NLP-12 OE) induced a hyperactive locomotion phenotype [60]. NLP-12 OE animals have a similar curvature (29.7^o^ ±1.4) to nRHO-1* animals (33.1^o^ ±0.5, p = 0.01) compared to wild type animals (17.4^o^ ± 0.3, p< = 0.0001) (Fig 6E). We analyzed the mRNA expression level of *nlp-12* in nRHO-1* animals and observed a significant increase in *nlp-12* mRNA in nRHO-1* animals with 28,954 ± 2211 copy no/reaction compared to wild type with 11,780 ± 354.2 copy no/reaction (p< = 0.0001) (Fig 6F). However, *dat-1* mRNA levels in the NLP-12 OE animals (86.7 ± 8.4 copy no/reaction) were not significantly different from wild type animals with 60.8 ± 7.0 copy no/reaction (p = 0.979) (Fig 6G). These data suggest that although nRHO-1* did significantly increase *nlp-12* expression the over-expression of *nlp-12* alone does not increase *dat-1* expression. Since high expression of NLP-12 alone does not increase *dat-1* expression we sought to determine whether any neuropeptides were involved in regulating *dat-1* expression, by using mutants of two proteins required for the priming and the exocytosis of neuropeptide loaded dense core vesicles, protein kinase C (PKC-1) and the CAPS-1 protein UNC-31 [61,62]. We analysed *dat-1* mRNA expression levels in a gain of function PCK-1 mutant (*pkc-1(gf) nuIs131*), which has elevated levels of neuropeptide release and is hypersensitive to aldicarb indicating a hyperactive locomotion phenotype [61], and also in the UNC-31 mutant (*unc-31(e928)*), which has reduced neuropeptide release and is lethargic [62,63] (Fig 6E). These mutants, however, did not show significant changes in *dat-1* mRNA expression levels (*pkc-1(gf)* 87.4 ± 17.3 and *unc-31(e928)* 129.7 ± 11.1 copy no/reaction) compared to wild type animals with 60.8 ± 7.0 copy no/reaction (p> = 0.05) (Fig 6G). We also analysed nRHO-1*;*unc-31(e928)* double mutants to see if reduced neuropeptide release caused by the *unc-31* mutation would decrease the increased *dat-1* expression caused by nRHO-1*. We observed a slight decrease in *dat-1* mRNA expression in nRHO-1*;*unc-31(e928*) animals with 167.4 ± 30.4 copy no/reaction when compared to nRHO-1* animals with 231.1 ± 30.9 copy no/reaction however this decrease was not significant (p = 0.294) (Fig 6G). The limitation of using the *pkc-1*(gf) and the *unc-31* mutants is that we are targeting general neuropeptide release and therefore cannot distinguish between the approximately 250 individual neuropeptides that have been identified to date, many of which have opposing effects on behaviour [64]. Therefore our data do not fully rule out the possibility that a specific set or single neuropeptide transmits the signal from cholinergic nRHO-1* expressing cells to dopaminergic neurons to upregulate *dat-1*.

### Is *dat-1* upregulation required for nRHO-1* modulation of locomotion?

**Fig 7:**
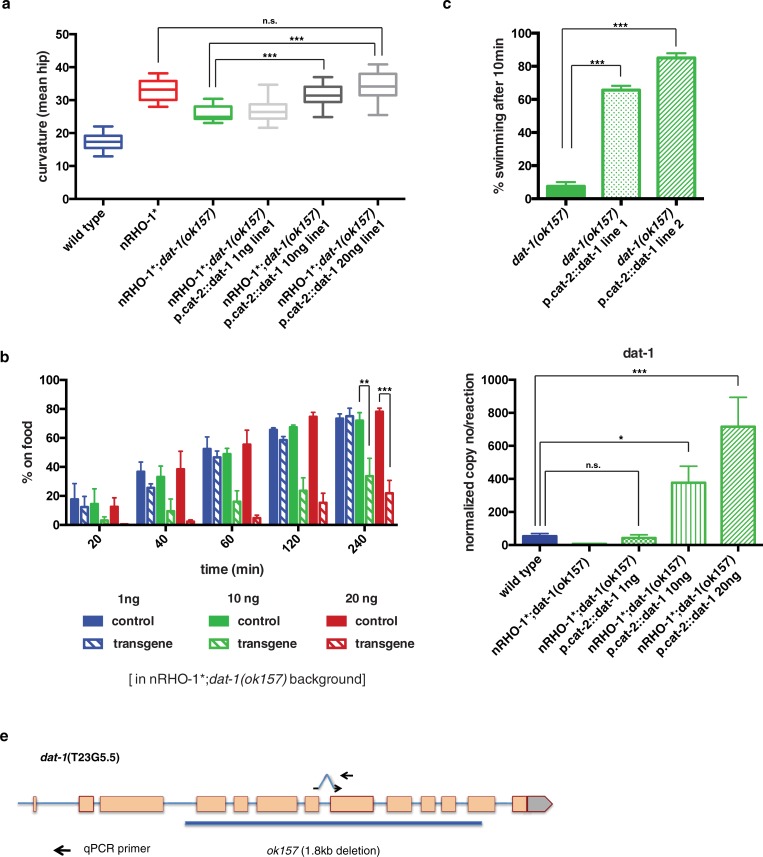
Increased *dat-1*-levels essential for nRHO-1* mediated loopy locomotion. **a** Worm tracking data of body curvature of control animals (wild type, nRHO-1* or nRHO-1*;*dat-1(ok157)* the same data used in Figs 2B, 3A, 4A and 6C) versus nRHO-1*;*dat-1(ok157)* expressing increasing concentrations of an RHO-1* independent *dat-1*-rescuing construct (p.cat-2::*dat-1*). One line of each concentration is shown as representative from two lines. Statistical comparisons were performed using one-way ANOVA and presented as mean hip ± SEM with Tukey’s multiple test correction. Statistical significance indicated as (***P < 0.001, n.s. = not significant), n = 70, 32, 15, 19, 20, and 20 animals respectively. **b** Dispersal assay as described in materials and methods. Genotypes are color-coded as indicated below the panel. nRHO-1*;*dat-1(ok157)* expressing increasing concentrations of an RHO-1* independent *dat-1*-rescuing construct (p.cat-2::*dat-1*). One line of each *dat-1*-rescuing construct concentration is shown as representative from two lines. Data presented as the mean percentage of animals on food at indicated time points ± SEM. Statistical comparisons were performed using two-way ANOVA with Tukey’s multiple test correction. Statistical significance indicated as (**P ≤ 0.01–0.001; ***P < 0.001), n = 3 experiments (~200 animals per assay). **c** RHO-1* independent *dat-1*-rescue construct p.*cat-2*::*dat-1* rescues SWIP of L4 stage *dat-1(ok157)* animals at low injection concentrations of 1ng/μl. Data presented as mean percentage ± SEM of animals swimming in water after 10 minutes. Statistical comparisons were performed using one-way ANOVA with Tukey’s multiple test correction. Statistical significance indicated as (***P < 0.001), n = 3 experiments (10 animals per assay). **d** Expression levels of *dat-1* in control (wild type) animals and non-injected control strain nRHO-1*;*dat-1(ok157)* compared to three rescue lines with increasing plasmid concentrations of *dat-1* under *cat-2* promoter (p.*cat-2*::*dat-1*) determined by quantitative PCR. Data presented as mean normalized copy number per reaction ± SEM. Statistical comparisons were performed using one-way ANOVA with Tukey’s multiple test correction. Statistical significance indicated as (*P≤0.05–0.01, ***P ≤ 0.001, n.s. = not significant), n = 6, 4, 4, 5, and 4 independent samples for each genotype respectively. **e** Schematic of the *dat-1* gene with the *ok157* allele indicated. qPCR primer set for *dat-1* within the *ok157* deletion shown as arrows.

We next investigated whether the transcriptional upregulation of *dat-1* expression was required for nRHO-1* signalling to drive locomotion. We utilized our finding that expression of the dopaminergic neuron specific *cat-2* gene is not upregulated by nRHO-1* (Fig 6B) and expressed *dat-1* from the *cat-2* promoter to generate a rescue construct in which *dat-*1 is no longer regulated by RHO-1 signalling. This construct was injected into nRHO-1*;*dat-1(ok157)* animals in a series of increasing concentrations, with the expectation that these transgenes would express *dat-1* in proportion to the increasing copy number. Our model is that for nRHO-1* to fully trigger changes in locomotion it must also reduce dopamine signalling. We predicted that in nRHO-1*;*dat-1(ok157)* animals with a transgene expressing low levels of *dat-1* mRNA there would be insufficient DAT-1 protein to inhibit dopamine signalling and that despite the enhanced RHO-1 signalling, these animals would not display a loopy locomotion phenotype. We observed that nRHO-1*;*dat-1(ok157)* animals with the lowest copy number of *dat-1* still showed suppression of curvature with 26.8° ±0.7 compared to 25.8° ±0.6 for nRHO-1*;*dat-1(ok157)* animals (p = 0.9931) and increased dispersal at 4 hours with 75.0% ±5.5 compared to 73.4% ±3.2 in nRHO-1*;*dat-1(ok157)* animals (p = 09987) (Fig 7A and 7B, Figure a and b in S4 Fig, S9 Movie). As we increased *dat-1* expression, by increasing the plasmid concentration, we observed a concomitant increase in curvature and decrease in dispersal (Fig 7A and 7B, Figure a and b in S4 Fig, S10 and S11 Movies). Animals injected with 10ng or 20ng of p.*cat-2*::*dat-1* increased the curvature of nRHO-1*;*dat-1(ok157)* animals significantly from 25.8° ±0.3 to 31.5° ±0.7 and 34.3° ±0.9 (both p< = 0.0001). At the highest level of *dat-*1 expression, 20ng of p.*cat-2*::*dat-1*, the curvature of the nRHO-1*;*dat-1(ok157)* animals equaled the curvature of nRHO-1* animals (33.1° ±0.5, p = 0.9162) (Fig 7A). The effect of increasing *dat-*1 expression on dispersal also matched our prediction as nRHO-1*;*dat-1(ok157)* animals containing transgenes created using 10ng and 20ng of p.*cat-2*::*dat-1* plasmid decreased dispersal to 33.7% ±12.2 (p = 0.0019) and 21.9% ±8.8 (p = 0.0001) respectively compared to non-transgenic animals of the same line (Fig 7B). Moreover, the dispersal behaviour of the 20ng rescue line was no longer significantly different from that of the hyperactive nRHO-1* animals (7.3% ±4.2, p = 0.2094) (Fig 2C).

The interpretation of these data depends on our assumption that the copy number of the transgenes is proportional to the expression of *dat-1* from these transgenes and that the 1ng of p.*cat-2*::*dat-1* produces a physiologically relevant amount of *dat-1* protein. We measured *dat-1* mRNA levels using *dat-1* primers that anneal within the *dat-1(ok157)* deletion (Fig 7D and 7E); this ensures that we measure transcripts only from the transgene and avoid any mRNA expression from the defective endogenous *dat-1* gene. In the absence of any transgene *dat-1* mRNA levels in nRHO-1*;*dat-1(ok157)* were 7.2 ±0.3 copy no/reaction. In nRHO-1*;*dat-1(ok157)* animals transgenes made with 1ng, 10ng, and 20ng of p.*cat-2*::*dat-1* plasmid the expression of *dat-1* was 43.1 ±19.2 (p = 0.99), 377.0 ±100.3 (p = 0.029), and 715.9 ±178 (p< = 0.0001) (Fig 7D), indicating that the increase in transgene concentration results in a proportional increased in dat-1 expression. The expression from the 1ng of p.*cat-2*::*dat-1* transgene was 43.1 ±19.2 which is very similar to that of wild type *dat-1* expression levels 53.5 ±15.9 (p = 0.99) (Fig 7D). Injection of 1ng of p.*cat-2*::*dat-1* plasmid was able to rescue the previously described swimming induced paralysis (SWIP) phenotype of the *dat-1(ok157)* animals [65,66] (Fig 7Cc), indicating that these transgenes do express *dat-1* mRNA at a level that produces sufficient functional DAT-1 protein to reduce this phenotype. However, the transgenes in the SWIP assays are independent from the 1ng of p.*cat-2*::*dat-1* transgene in the nRHO-1*;*dat-1(ok157)* animals and may not produce identical amounts of protein. Our data therefore suggest that *dat-1* expression levels correlate with the increased ability of RHO-1 signalling to trigger locomotion with high curvature and a failure to disperse, however we have not fully confirmed this. Taken together our data suggest that *dat-1* expression is necessary for RHO-1 signalling to drive locomotion and that *dat-1* is a new component of a neuronal RHO-1 signalling pathway that regulates locomotion.

## Discussion

In this study we describe our finding that the *C*. *elegans* ortholog of RhoA, RHO-1, modulates locomotory behaviour by repressing dopamine signalling through the upregulation of the dopamine re-uptake transporter DAT-1. The most striking phenotype of expressing constitutively active RHO-1 in cholinergic neurons (nRHO-1*) is a switch from normal sinusoidal locomotion to a hyperactive loopy locomotion [18]. This phenotype was quantified in two ways: worm tracking to analyse the body curvature and dispersal assay to analyse the animal’s ability to coordinate their locomotion towards a food source. nRHO-1* animals have increased body curvature with much larger body bends and they cannot coordinate their locomotion and fail to disperse, together we refer to this as loopy locomotion. The ability of nRHO-1* to trigger loopy locomotion is strongly reduced in two separate *dat-1* mutant alleles, *nz99* and *ok157*. We rescued this phenotype by expressing a wild type *dat-1* transgene suggesting that DAT-1 has a role in RHO-1 mediated loopy locomotion. The *dat-1(ok157)* also reduced RHO-1* loopy locomotion following heat shock of hs::RHO-1* animals, indicating this is not a strain specific effect. The reduction of loopy locomotion is not 100%, which could hint to *dat-1* and nRHO-1 acting in parallel pathways or, more likely, due to the nature of activated RHO-1* signalling being very complex and involving multiple pathways.

DATs negatively regulate dopamine signalling by removing dopamine from the extracellular space reducing the concentration and spread of dopamine released from the dopaminergic neurons [21]. DAT mutations are predicted to increase extracellular dopamine abundance and signalling. However, it is also possible that DAT-1 acts as a dopamine-gated depolarizing ion channel or that chronically high levels of dopamine cause adaption such that dopamine signalling could be decreased in *dat-1* mutant animals [67,68]. To test if increased dopamine signalling is involved in the suppression of nRHO-1* loopy locomotion we used a *cat-2* mutant background, in which dopamine synthesis is selectively reduced. The loopy locomotion phenotype was no longer suppressed by the *dat-1* mutation in a nRHO-1*;*dat-1;cat-2* triple mutant background supporting our prediction that dopamine signalling is involved. In contrast, if *dat-1* mutants suppressed nRHO-1*-mediated locomotion due to chronically decreased dopamine signalling or if DAT-1 functions as a dopamine-activated ion channel, the effect of the *cat-2* mutation would have been minor. Furthermore, we exposed nRHO-1* single mutant animals, which have increased levels of *dat-1* expression, to exogenous dopamine and observed a suppression of their loopy phenotype. These data indicate that loss of *dat-1* results in an increase in dopamine signalling which supresses the loopy locomotion phenotype of nRHO-1*. We reasoned that if increased dopamine were responsible for antagonizing nRHO-1* then this would require a dopamine receptor. There are four well characterized dopamine receptors in *C*. *elegans*, DOP-1-4 [48]. Mutations in *dop-1*, and partially *dop-4*, reversed *dat-1* suppression of the loopy locomotion in nRHO-1 animals. No change in expression was observed for any of the DOP receptors in the nRHO-1* animals. Taken together these results suggest *dat-1* suppression of nRHO-1* acts through increased dopamine signalling and unlikely through the reported DAT-1 ion channel function or a change in dopamine receptor signalling [67]. The effect of the *dat-1* mutation on dopamine signalling is likely to be complex with changes that will include both the strength of the dopamine signal received by a cell and the number of cells that respond to dopamine as well as other changes [39]. Our data indicate that mutations in *dat-1* cause an increase in dopamine signalling via the DOP-1 receptor that prevents nRHO-1* from triggering the loopy locomotion phenotype. This result is somewhat surprising as DOP-3 receptors have been shown to be involved in the basal slowing response upon encountering food, paralysis on exogenous dopamine and swimming induced paralysis [39,65]. Allen *et al* reports that DOP-3 signalling reduces ACh-release from motor neurons whereas DOP-1 signalling stimulates ACh-release [40]. nRHO-1*;*dat-1* double mutants despite showing a clear reduction in loopy locomotion remain hypersensitive to aldicarb indicating no change in ACh release from motor neurons. DOP-1 receptors most likely act in an alternative pathway to repress loopy locomotion. It has been shown recently that RHO-1 acts to stimulate NCA channel activity to regulate locomotion [16], and that dopamine signalling acts via DOP-3 receptors to antagonize NCA-1 channel activity [17]. The mutation in *dop-3*, however, had no effect on the locomotion of nRHO-1*;*dat-1(ok157)* animals, therefore the underlying mechanism is likely to be a different one.

In *dat-1* mutants the absence of DAT-1 prevents nRHO-1* from triggering loopy locomotion so how does nRHO-1* override the antagonistic dopamine signalling to trigger loopy locomotion in animals with a functioning *dat-1* gene? Exogenously administered dopamine reduced loopy locomotion in nRHO-1* animals leading to paralysis, however, the nRHO-1* animals paralysed at a much slower rate in comparison to wild type animals. One interpretation of these data could be that there is reduced dopamine abundance at its site of action in the nRHO-1* animals. We argue that this is achieved by increasing the levels of the dopamine reuptake transporter DAT-1 in the nRHO-1* animals. We observed that nRHO-1* increased expression of an p.*dat-1*::mCherry::DAT-1 fusion protein and showed that transcription from the endogenous *dat-1* gene was increased in nRHO-1* animals. Genes required for the synthesis and release of dopamine from dopaminergic cells share transcriptional control elements with *dat-1* [52,69]. Of these only *dat-1* and *cat-2* are expressed exclusively in dopaminergic cells and transcription of *cat-2* according to our qPCR data was unaffected by nRHO-1*. We also analysed the expression levels of other dopamine pathway genes such as the dopamine receptors *dop-1-4* and a general neuronal gene expressed in dopaminergic neurons, *snb-1*, none of which showed significant alteration in nRHO-1* animals. These data indicate that the effects of nRHO-1* on dopaminergic cells did not cause a general increase in transcription, suggesting that RHO-1 acts via DAT-1 to decrease dopamine signalling. We investigated whether the upregulation of *dat-1* was necessary for the induction of loopy locomotion, by exploiting the observation that *cat-2* mRNA level did not significantly increase in response to nRHO-1* based on our qPCR data. We made a nRHO-1* independent *dat-1* transgene using the *cat-2* promoter (p.*cat-2*::*dat-1*). nRHO-1*;*dat-1(ok157)* animals carrying these transgenes showed a correlation between increased expression of *dat-1* through higher transgene concentrations and increased loopy locomotion. These data lead us to hypothesize that nRHO-1* increases the transcription of the *dat-1* gene in order to trigger loopy locomotion. However, we recognize the caveat that the low copy number transgene, even though expressed at the similar level to the endogenous *dat-1* in wild type animals, might have been too low to rescue the nRHO-1* phenotype as the function of an different low copy number plasmid was tested in the SWIP assay. Additionally, the expression level of *dat-1* required to rescue the SWIP phenotype might be different to the one required to rescue the locomotion phenotype in a nRHO-1*;*dat-1(ok157)* double mutant. However, active RHO-1 was not able to increase expression levels of *dat-1* when placed under the *cat-2* promoter. As *dat-1* mutations can only suppress nRHO-1* in the presence of dopamine synthesis and signalling, we interpret our data as follows: RHO-1 increases *dat-1* transcription resulting in increased DAT-1 protein at the cell surface and consequently in a reduction of extracellular dopamine. Our data also suggest that without a DAT-1-mediated reduction in dopamine signalling the RHO-1 signalling pathway is severely reduced in its ability to regulate locomotion. These data indicate that we have identified an additional branch of the neuronal RHO-1 signalling pathway, which modulates locomotion by regulating the expression of DAT-1 and thus dopamine signalling.

The increase in *dat-1* expression is not just an indirect consequence of loopy locomotion as nRHO-1* increases expression from the non-functional *dat-1(ok157)* gene in non-loopy nRHO-1*;*dat-1(ok157)* animals. The *dat-1(ok157)* allele cannot produce functional DAT-1 protein due to a deletion in the *dat-1* 3’ end, however, the *dat-1(ok157)* deletion leaves the *dat-1* promoter and transcription start sites intact and we observed an increase from this faulty *dat-1* gene in the non-loopy nRHO-1*;*dat-1(ok157)* animals. Moreover, *dgk-1* mutants do not increase *dat-1* expression despite having a similar body curvature as nRHO-1*;*dat-1(ok157)*, indicating that nRHO-1* mediated increases in *dat-1* expression are not an indirect effect of increased body curvature during loopy locomotion. How does RHO-1 regulate *dat-1* expression when the cells expressing nRHO-1* and *dat-1* do not overlap? Although p.unc-17 is a promoter widely used to drive cholinergic neuron specific expressions of proteins [17,18] at this moment we cannot exclude that p.unc-17::nRHO-1* may be expressed at a low level in cells other than cholinergic neurons such as the same cell as *dat-1*. RhoA has been shown to regulate DAT-internalization in mouse midbrain slices [31], and DAT-surface availability in *C*. *elegans* [43] cell autonomously linking RHO-1 to DAT function. However, our hypothesis is that the cholinergic cells which express nRHO-1* release a RHO-1-regulated signal that acts on the dopaminergic cells to non-autonomously regulate *dat-1* expression. The signal is unlikely to be ACh, although we cannot fully exclude the possibility that increased ACh release is necessary for *dat-1* expression. nRHO-1* induces the release of the neurotransmitter ACh from synaptic vesicles [18] but, ACh release is also stimulated in *dgk-1* mutants [18] and *pkc-1*(gf) animals [61] neither of which show increased *dat-1* expression. With RHO-1 playing a role in the regulation of synaptic vesicle release [18] we investigated the possibility that RHO-1 signalling could also regulate dense core vesicle release and therefore the release of neuropeptides. A number of neuropeptides have been shown to regulate locomotion [54–56]. The neuropeptide NLP-12 modulates dopamine signalling via DOP-1 and over-expression of *nlp-12* induces a hyperactive locomotion phenotype [60]. Although NLP-12 OE animals have a very similar loopy locomotion as nRHO-1* animals and we show that nRHO-1* significantly increases *nlp-12* expression, *dat-1* expression however was not significantly increased in the NLP-12 OE animals. These data indicate that high levels of NLP-12 do not increase *dat-1* expression, but these data do not fully exclude the involvement of nlp-12 in this RHO-1 signalling pathway. The use of an *nlp-12* loss of function mutation may shed more light on its involvement. Targeting general neuropeptide release via UNC-31 or PKC-1 mutants did not deliver conclusive results on whether neuropeptides are involved in our pathway. We believe that one or more of the over 250 distinct neuropeptides [64] would be a good candidate for the RHO-1 mediated regulation of *dat-1* expression and targeting individual neuropeptides represents a future task in order to understand how RHO-1 regulates *dat-1* expression to control locomotion.

In this study, we propose that changes in *dat*-1 expression affect the behaviour of *C*. *elegans*, so how likely is it that changes in *Dat* expression can cause behavioural changes in other species? Mice heterozygous for a *Dat* deletion display changes in dopamine levels intermediate between that of wild type and full knockout mice [70]. In rats iron deficiency reduced *Dat* expression and this correlated with changes in motor behaviour [71]. In humans DAT polymorphisms can alter *DAT* expression and variants associated with human mental diseases can alter transcription over a 3 fold range, approximately the same range we observed nRHO-1* to alter *dat-1* expression [72]. The contribution of these human DAT polymorphisms to mental disorders is being intensely studied with the clearest connection being that between changes in *DAT* expression and ADHD, however, linkage between *DAT* alleles and a large range of mental disorders or general behavioural traits are being reported [23,73]. The *C*. *elegans* RHO-1/DAT-1 pathway increases *dat-*1 expression almost fourfold, from the information above such changes in humans would alter many brain functions such as changes in mood, motivation, movement, and cognition. If the RHO-1/DAT-1 pathway is conserved in humans then any defects in this pathway could have effects ranging from subtle changes in behaviour to severe mental disorders. Drugs capable of targeting such a DAT regulatory pathway could have clinical applications for treatments of the wide range of mental conditions that DATs are increasingly being implicated in.

## Materials and methods

### Strains

All strains were cultivated at 20°C unless otherwise stated and were maintained as described previously [74]. N2 (wild-type), CB1112 *cat-2(e1112)*, RM2702 *dat-1(ok157)*, LX636 *dop-1(vs101)*, LX702 *dop-1(vs105*), LX703 *dop-3(vs106)*, FG58 *dop-4(tm1392)*, PS2627 *dgk-1(sy428)*, DA509 *unc-31(e928)* strains were obtained from the Caenorhabditis Genetics Center (University of Minnesota).

BY602 *dat-1(ok157)*;*cat-2*(e1112), QT1152 *dat-1(ok157)*;*dop-2(vs105)*, QT1153 *dat-1(ok157)*;*dop-1(vs101)*, QT1154 *dat-1(ok157)*, *dop-3(vs106)* a kind gift from Randy Blakely (Department of Psychiatry, Vanderbilt University School of Medicine, Nashville, TN).

NLP-12 OE *nlp-12(OE) (ufIs104* (*p*.*nlp-12*::*nlp-12* genomic locus)*)* a kind gift from Michael Francis (Department of Neurobiology, University of Massachusetts Medical school, Worcester, MA).

KP1380 *pkc-1(gf) (nuIs131(p*.*myo*::*gfp;p*.*unc-17*::*pkc-1B(A160E))* a kind gift from Derek Sieburth (Zilkha Neurogenetic Institute, Keck School of Medicine, University of Southern California, LA, CA).

QT309 nzIs1* (heatshock::rho-1*;*p*.*ttx-3*::GFP) *nzIs29 (p*.*unc-17*::rho-1*;p.*unc-122*::GFP) double mutant was made from crossing strains with the previously described transgenes [18], QT677 *nzIs1;nzIs29;nz99* (isolated from a standard EMS genetic screen), QT1125 *nzIs29;dat-1(ok157)*, QT1331 *nzIs1;dat-1(ok157*), QT1343 *dat-1(ok157);dop-4(tm1392*), QT1293 *nzIs29;dat-1(ok157);dop-1(vs101)*, QT1421 *nzIs29;dat-1(ok157);dop-2(vs105)*, QT1335 *nzIs29;dat-1(ok157);dop-3(vs106)*, QT1422 *nzIs29;dat-1(ok157)*,*dop-4(tm1392)*, QT1441 *nzIs29;dat-1(ok157);cat-2(e1112)*, QT776 *nzIs29;unc-31(e928)*.

### Transgenic strains

Transgenic strains (listed as QT) were isolated by microinjection of 20 ng/ml plasmid unless otherwise stated, together with *p*.*acr-2*::mCherry (SJN445) at 50 ng/mL (a gift of O. Hobert, Columbia University, New York, NY) or p.*snb-1*::GFP (QT#237) at 50 ng/μl as a marker. QT#42 heat shock hs::RHO-1* (G14V) [75]. *nzIs1* contains heatshock::RHO-1*;*p*.*ttx-3*::*gfp*, *nzIs29* contains *(p*.*unc-17*::RHO-1*;*p*.*unc-122*::*gfp)*

QT1412 *nzIs29;dat1(ok157);nzEx690*[SJN617(genomic_dat-1);SJN445(*p*.*acr-2*::mCherry)] line 2, QT1413 *nzIs29;dat1(ok157)*;*nzEx691*[SJN617(genomic_dat-1);SJN445(*p*.*acr-2*::mCherry)] line 1, QT1430 *nzIs29;dat-1(ok157);dop-1(vs101)*;nzEx701[fosmid WRM069cF02;QT#237(p.*snb-1*::GFP)] line 2, QT1435 *nzIs29;dat-1(ok157);dop-1(vs101)*;*nzEx704*[fosmid WRM069cF02;QT#237(p.*snb-1*::GFP)] line 1, QT1436 *dat-1(ok157)*;*nzEx705*[QT#42(hs::RHO-1*(G14V));QT#237(p.*snb-1*::GFP);SJN717 1ng] line 2, QT1437 *dat-1(ok157);nzEx706*[QT#42(hs::RHO-1*(G14V));QT#237(p.*snb-1*::GFP);SJN717 1ng] line 1, QT1419 *nzIs29;dat1(ok157)*;*nzEx697*[SJN717(1ng)(*p*.*cat-2*::*dat-1*);QT#237(p.*snb-1*::GFP)] line 1, QT1423 *nzIs29;dat1(ok157)*;*nzEx698*[SJN717(1ng)(*p*.*cat-2*::*dat-1*);QT#237(p.*snb-1*::GFP)] line 2, QT1416 *nzIs29;dat1(ok157)*;*nzEx694*[SJN717(10ng)(*p*.*cat-2*::*dat-1*);QT#237(p.*snb-1*::GFP)] line 2, QT1417 *nzIs29;dat1(ok157)*;*nzEx695*[SJN717(10ng)(*p*.*cat-2*::*dat-1*);QT#237(p.*snb-1*::GFP)] line 1, QT1414 *nzIs29;dat1(ok157)*;*nzEx692*[SJN717(20ng)(*p*.*cat-2*::*dat-1*);QT#237(p.*snb-1*::GFP)] line 2, QT1415 *nzIs29;dat1(ok157)*;*nzEx693*[SJN717(20ng)(*p*.*cat-2*::*dat-1*);QT#237(p.*snb-1*::GFP)] line 1, QT1291 nzEx649[QT#42(1ng)(hs::RHO-1*(G14V));SJN628(p.*dat-1*::mCherry::dat-1;QT#237(p.*snb-1*::GFP)], QT1437 *nzIs29;nzEx649*, QT1438 *nzEx649*.

### Plasmids

Plasmids (listed as QT# or SJN) were constructed by standard techniques, and verified by sequencing.

SJN617 pBS *dat-1* genomic fragment starting 722bp 5’ of the start ATG and 918bp 3’ of the STOP of *dat-1*a was amplified by PCR with a SacI site added 5’ and a KpnI site added 3’ and subcloned into pBluescript SK.

SJN717 *p*.*cat-2*::*dat-1* genomic, an EagI site 40bp 5’ of the *dat-1* start ATG was used to swap out the promoters. An 852bp fragment of *cat-2* starting 901bp 5’ and finishing 47bp 5’ of the *cat-2* start ATG was amplified with PCR with a SacI site 5’ and an EagI site 3’ and used to swap out the SacI-EagI fragment in the *dat-1* promoter of SJN617. SJN628 p.*dat-1*::mCherry::dat-1 A NotI site plus one base GCGGCCGCc was inserted immediately 3’ to the *dat-1*a start ATG in SJN617. Into this NotI site was subcloned mCherry amplified with flanking NotI sites such that it was in frame with *dat-1* with 3 extra Alanines added at the N and C terminus by the flanking NotI plus a base sites.

### Induction of heat-shock-inducible transgenes

In all cases, expression from the heat-shock promoter was achieved using two rounds of heat shock for 60 min at 30°C, separated by 30 min at 20°C, followed by recovery for 30 min at 20°C [18].

### Fluorescence microscopy

Transgenic lines were mounted on agarose pads in paralyzing agent (10 mg/ml 2,3-Butanedione monoxime, Sigma) and imaged on a Perkin Elmer Ultraview Vox spinning disc confocal using a 100x oil lens. Laser images and exposures times were the same per channel and images were obtained and processed to give maximum intensity projections of a z-series using Perkin Elmer Volocity 6.3 software. Several control and mutants animals were observed in the same session, and these sessions were repeated on different days.

### Aldicarb assay

Sensitivity to 1 mM aldicarb (Greyhound Chromatography) on NGM plates in the presence of food was determined by analysing the onset of paralysis as described previously [53]. For each experiment, 30 animals were tested, and each experiment was repeated at least in triplicate.

### Dopamine locomotion assay

Assays were performed as described in [39]. For the assay, approx. 30 young adult animals were transferred to 1.7% agar plates containing 2 mM glacial acetic acid plus or minus 15 mM dopamine, and the number of paralysed animals were scored every 10 min for 100 min. Animals were scored as paralysed if they no longer responded to harsh touch and each experiment was repeated at least in triplicate. For Worm tracking young adult animals were individually transferred to dopamine plates plus or minus 20 mM dopamine, incubated for 20 min, and then recorded on the Worm Tracker 2.0.4 system. A minimum of 16 animals were tracked per condition.

### Calculation of curvature

The mean curvature of the hip (Fig 1C) was analysed by tracking single worms using the Worm Tracker 2.0.4 system as developed by the Schafer Lab [46]. In summary, 1-day-old adults were transferred to low peptone tracking plates, seeded the day before with a full lawn of OP50, and individually tracked following a 30 min acclimatization period. Animals were tracked for 5 min and at least 12 animals were assayed over different days for each strain, with each batch containing wild type animals. The videos were analysed using Worm Analysis Tool Box version 3 and Matlab (Matlab script see S1 Script). For clarity one point on the worm, mean hip (point 4), was chosen to represent alterations in curvature on subsequent strains. This was done firstly as a means to cut down on the presented data and mean hip was specifically chosen due to potentially less interference from head movements when foraging [76] which, could affect point 1 and 2. Point 3 could potentially be affected by the swollen vulva phenotype of nRHO-1* animals [75] and point 5 could be affected by defecation and/or tail swelling another phenotype of nRHO-1* animals [77].

### SWIP assay

Swimming induced paralysis (SWIP) assays were performed as described in [66]. In summary 10 L4 animals were transferred to a 45ul spot of dH2O and scored for SWIP after 10 min. We defined paralysed animals in our studies as absence of movement for at least 20 sec. The assay was repeated at least in triplicate for each strain.

### Dispersal assay

Assays were performed as described in [47]. In summary OP50 was seeded at the edge of a 10 cm NMG plate 24 hours before assay. A plate of non-starved adult animals were washed 3x in M9 buffer (animals were allowed to settle on ice, by gravity, between each wash, as centrifugation altered dispersal of animals). Approximately 200 animals were added to the centre of the plate and timed once the M9 buffer had been absorbed. The number of animals on food, were scored at 20, 40, 60, 120 and 240 minutes and the total number of adults were scored at the end of the assay. The assay was repeated at least in triplicate for each strain on different days to minimise batch effect (Fig 1D).

### Quantitative PCR

RNA was isolated using Qiagen RNeasy mini kit following the manufacturers protocol. At least 3 independent samples were used per genotype. qPCR was performed and analyzed by qStandard (*www.qstandard.co.uk*, 61 Wolmer Gardens, HA8 8QB Edgware UK). Primer sequences are as follows; *dat-1* set 1 forward agttggtgcctacagacgat reverse tccagcaagagaacagtggtc (Fig 6D), *dat-1* set 2 (primers that anneal within the *dat-1(ok157)* deletion) forward gaaatgctcaagagaccatcgg reverse tcggagttctcatggcatactc (Fig 7E), *cat-2* set forward caacaacggatccacgacat reverse gcctatctcgtcaccaaact, *nlp-12* set forward gattaccggccacttcagttc reverse cgttttccgaactgcaatgga. Three reference genes were used based on unusually stable expression levels with relatively little variation between adults, dauers, and L3 larvae (qStandard): Y45F10D.4 forward aagcgtcggaacaggaatc reverse gagtcgacgataacggaaaaa, pmp-3 (C54G10.3.1) forward tcgctgaaacaattccatga reverse atggtcccttcacgacattg, cdc-42 (R07G3.1), forward tatgtgccgacagtcttcgacaatta, reverse ctctatcgtatccacagaccga. The data were normalized by dividing the copy number per reaction by the normalization factor of three reference genes from geNorm. geNorm (https://genorm.cmgg.be/) is a popular algorithm to determine the most stable reference (housekeeping) genes from a set of tested candidate reference genes in a given sample panel. From this, a gene expression normalization factor can be calculated for each sample based on the geometric mean of a user-defined number of reference genes.

### Screen and whole genome sequencing

nRHO-1* animals were mutagenized using a standard *C*. *elegans* EMS protocol. 1930 haploid genomes were screened, 116 strong suppressors of loopy locomotion (15 were unreproducible, 15 contained inactivated nzIs29 transgene) so 86 suppressor mutants were identified. The QT677 non-loopy nRHO-1*;*nz99* animals were detected by eye, singled and their progeny were shown to be non-loopy. This non-loopy phenotype was maintained following heat shock activation of the second integrated nRHO-1* transgene, *nzIs1*. QT677 was chosen as a neuronal-specific suppressor mutant because while it suppressed loopy locomotion driven by both transgenes, it failed to suppress the sterility phenotype of hs::RHO-1*, which is thought to have a non-neuronal origin [75]. Whole genome sequencing (WGS) and bioinformatics of un-backcrossed nRHO-1*;*nz99* animals was performed by GeneService (http://www.sourcebioscience.com/) using Illumina 76bp paired ends sequencing that resulted in an average coverage of greater than 10x. The WGS data of the QT677 strain (nRHO‐1*; *nz99*) was compared against the reference nRHO‐1* strain and 2809 mutations were identified, 1278 of which were induced by EMS. These 1278 mutations led to 42 protein coding altering mutations identified by analysis with MAQSeq [78] (S1 Table).

### Statistical analysis

All raw data is provided as supplementary information (S1 Dataset). Statistical analysis was performed as specified in the figure legends, using either an unpaired two-tailed t-test, one-way ANOVA with Tukey’s multiple test correction or two-way ANOVA with Tukey’s multiple test correction using GraphPad Prism®. P values are indicated as follows * ≤ = 0.05–0.01 ** ≤ = 0.01–0.001 and *** ≤ = 0.001.

## Supporting information

S1 Fig *dat-1(ok157)* mutation suppresses hs::RHO-1* locomotion and *dat-1(ok157)* versus wild type animals body curvature. **a** Worm tracking data of body curvature of wild type, *dat-1(ok157)*, hs::RHO-1* and hs::RHO-1*;*dat-1(ok157)* animals following heat shock. Statistical comparisons were performed using one-way ANOVA and presented as mean hip ± SEM with Tukey’s multiple test correction. Statistical significance indicated as (***P ≤ 0.001, n.s. = not significant), n = 9, 10, 5 and 11 animals respectively).
**b** Worm tracking data of body curvature at 5 body points within the worm (as shown in Fig 1C) comparing control animals (wild type the same data used in Fig 2B) to *dat-1(ok157)* animals. Statistical comparisons were performed using one-way ANOVA and presented as mean curvature ± SEM with Tukey’s multiple test correction. Diamonds indicate outliers. Statistical significance indicated as (n.s. = not significant), n = 70 and 14 animals respectively.
(EPS)

S2 Fig Locomotion of control strains *dop-1-4* singles and double with *dat-1(ok157)* mutation. **a** Worm tracking data of body curvature of control animals (wild type, *dat-1(ok157)* the same data used in Figs 2B, 3A, 4A, 6C and 7A), and four dopamine receptor single or dopamine receptor *dat-1(ok157)* double mutants. Statistical comparisons were performed using one-way ANOVA and presented as mean hip ± SEM with Tukey’s multiple test correction, n = 70, 14, 15, 15, 12, 16, 13, 13, 13, and 14 animals respectively.
**b** Dispersal assay of control animals (*dat-1(ok157)* the same data used in Fig 2C), and *dop-4(tm1392)* single and *dat-1(ok157);dop-4(tm1392)* double mutants. Genotypes are color-coded as indicated right of the panel. Data presented as the mean percentage of animals on food at indicated time points ± SEM, n = 3 experiments (~200 animals per assay).
(EPS)

S3 Fig nRHO-1* does not affect the transcription levels of *dop-1-4* or *snb-1*. **a-e** Transcription levels of *dop-1*, *dop-2*, *dop-3*, *dop-4*, *and snb-1* in nRHO-1* animals compared to control strain (wild type), determined by quantitative PCR. Data presented as mean normalized copy number per reaction ± SEM. Statistical comparisons were performed using an unpaired two-tailed t-test. Statistical significance indicated as (n.s. = not significant), n = 3 independent samples for each genotype respectively.
(EPS)

S4 Fig Increased *dat-1*-levels are essential for nRHO-1* mediated loopy locomotion. **a** Worm tracking data of body curvature of control animals (wild type, nRHO-1* or nRHO-1*;*dat-1(ok157)* the same data used in Figs 2B, 3A, 4A, 6C and 7A) versus nRHO-1*;*dat-1(ok157)* expressing increasing concentrations of an RHO-1* independent *dat-1*-rescuing construct (p.cat-2::*dat-1*). Two lines of each concentration are shown. Statistical comparisons were performed using one-way ANOVA and presented as mean hip ± SEM with Tukey’s multiple test correction. Diamonds indicate outliers. Statistical significance indicated as (***P < 0.001), n = 70, 32, 15, 19, 17, 20, 16, 20 and 19 animals respectively.
**b** Dispersal assay as described in materials and methods. Genotypes are color-coded as indicated below the panel. nRHO-1*;*dat-1(ok157)* expressing increasing concentrations of an RHO-1* independent *dat-1*-rescuing construct (p.cat-2::*dat-1*). Two lines of each concentration are shown. Data presented as the mean percentage of animals on food at indicated time points ± SEM. Statistical comparisons were performed using two-way ANOVA with Tukey’s multiple test correction. Statistical significance indicated as (**P ≤ 0.01–0.001, ***P < 0.001, n.s. = not significant), n = 3 experiments (~200 animals per assay).
(EPS)

S1 Table Predicted protein coding changes in QT677 nRHO-1*;*nz99*. Average coverage was greater than 10X. Bold indicates mutation in *dat-1*. Whole Genome sequencing and bioinformatics were performed by GeneService (http://www.sourcebioscience.com/).
(DOCX)

S1 Movie Representative locomotion of wild type animals. (MP4)

S2 Movie Representative locomotion of nRHO-1* animals. (MP4)

S3 Movie Representative locomotion of nRHO-1*;*dat-1(ok157)* animals. (MP4)

S4 Movie Representative locomotion of *dat-1(ok157)* animals. (M4V)

S5 Movie Representative locomotion of nRHO-1*;*dat-1(ok157)*;genomic_*dat-1* rescue animals. (MP4)

S6 Movie Representative locomotion of nRHO-1*;*dat-1(ok157);cat-2(e1112)* animals. (MP4)

S7 Movie Representative locomotion of nRHO-1* animals exposed to 20mM dopamine. (MP4)

S8 Movie Representative locomotion of nRHO-1*;*dat-1(ok157);dop-1(vs101)* animals. (MP4)

S9 Movie Representative locomotion of nRHO-1*;*dat-1(ok157)* containing 1ng of p.*cat-2::dat-2* transgene line 1 animals. (MP4)

S10 Movie Representative locomotion of nRHO-1*;*dat-1(ok157)* containing 10ng of p.*cat-2::dat-2* transgene line 1 animals. (MP4)

S11 Movie Representative locomotion of nRHO-1*;*dat-1(ok157)* containing 20ng of p.*cat-2::dat-2* transgene line 1 animals. (MP4)

S1 Script Matlab script. Script used to analyse videos with Worm Analysis Tool Box version 3.
(M) Click here for additional data file.

S1 Dataset Raw data. All raw data from the study is provided as an Excel file supplementary information.
(XLSX)
