# Supplementary material for: Activation of RHO-1 in cholinergic motor neurons competes with dopamine signalling to control locomotion
Source: PLoS One. 2018 Sep 21;13(9):e0204057. doi: 10.1371/journal.pone.0204057 (PMC6150489; doi:10.1371/journal.pone.0204057)
Supplement: S1 Table — Average coverage was greater than 10X. Bold indicates mutation in dat-1. Whole Genome sequencing and bioinformatics were performed by GeneService (http://www.sourcebioscience.com/). (DOCX) [file pone.0204057.s005.docx]

| Chromosome | Position | Class | Description | Gene Name |
| --- | --- | --- | --- | --- |
| I | 1396032 | missense | GCA->ACA [Ala->Thr] | Y92H12BR.7 |
| I | 1649388 | frameshift | frameshift (indel, +1 base) | mab-20 |
| I | 5990322 | missense | GTT->GGT [Val->Gly] | C06A5.3 |
| I | 6514481 | missense | TTG->TTC [Leu->Phe] | glh-2 |
| I | 8624283 | missense | GGA->AGA [Arg->Thr] | fer-1 |
| II | 866038 | missense | AGA->ACA [Arg->Thr] | Y46B2A.3 |
| II | 1539427 | missense | CTC->TTC [Leu->Phe] | fbxb-44 |
| II | 2903202 | missense | GAG->GGG [Glu->Gly] | Y110A2AM.1 |
| II | 3797203 | missense | CAA->CCA [Gln->Pro] | Y8A9A.2 |
| II | 5079267 | missense | GGC->GCC [Gly-Ala] | dsh-2 |
| II | 5079269 | missense | TTT->TTG [Phe->Leu] | dsh-2 |
| II | 6759911 | frameshift | frameshift (indel, -4 base) | F41G3.2 |
| II | 7451225 | missense | ACG->ATG [Thr->Met] | Y9D1A.2 |
| II | 7866906 | missense | AGG->TGG [Arg->Trp] | ztf-17 |
| II | 11212543 | missense | CTT->CCT [Leu->Pro] | srap-1 |
| II | 12668766 | missense | GAT->GTT [Asp->Val] | rgs-4 |
| II | 12668767 | missense | GAT->GAG [Asp->Glu] | rgs-4 |
| III | 8068400 | missense | AGT->AGA [Ser->Arg] | ceh-26 |
| III | 8068401 | missense | ACT->GCT [Thr->Ala] | ceh-26 |
| III | 8358762 | missense | TCT->TTT [Ser->Phe] | rfp-1 |
| III | **9245321** | **Premature stop** | **GAG->TAG [Glu->stop]** | **dat-1** |
| III | 10437049 | missense | TGT->TAT [Cys->Try] | enu-3 |
| III | 12470206 | frameshift | frameshift (indel, 2 base) | Y49E10.29 |
| IV | 5849520 | missense | CAA->CAT [Gln->His] | sams-3 |
| IV | 6485407 | missense | AGA->AGT [Arg->Ser] | tyr-6 |
| IV | 7735094 | missense | GTT->GAT [Gly->Asp] | clx-1 |
| IV | 9770293 | missense | ATT->GTT [Ile->Val] | msp-78 |
| IV | 10441763 | missense | GTA->GGA [Val->Gly] | F13B12.3 |
| IV | 12931059 | missense | CAA->CGA [Gln->Arg] | C32H11.7 |
| V | 3326356 | missense | AGA->GGA [Arg->Gly] | C17B7.7 |
| V | 3326546 | missense | AAT->AAA [Asn->Lys] | C17B7.7 |
| V | 8706638 | missense | CCA->GCA [Pro->Ala] | T07F10.5 |
| V | 12866414 | missense | CAG->CCG [Gln->Pro] | ZC178.2 |
| V | 20211027 | missense | GAA->GGA [Glu->Gly] | Y113G7B.12 |
| V | 20211293 | missense | GAT->GAA [Asp->Glu] | Y113G7B.12 |
| X | 5700 | missense | GAA->AAA [Glu->Lys] | CE7X_3.1 |
| X | 1836429 | missense | AGT->AAT [Ser->Asn] | T26C11.2 |
| X | 11056036 | missense | AGA->AGT [Arg->Ser] | C09F12.2 |
| X | 11767391 | missense | TGC->TAC [Cys->Try] | ttx-3 |
| X | 11767392 | missense | TGC->GGC [Cys->Gly] | ttx-3 |
| X | 15748986 | missense | TCG->TTG [Ser->Leu] | ZK678.3 |
| X | 15748987 | missense | TCG->ACG [Ser->Thr] | ZK678.3 |
